# Accuracy of Estrogen and Progesterone Receptor Assessment in Core Needle Biopsy Specimens of Breast Cancer

**DOI:** 10.5812/ircmj.10232

**Published:** 2013-06-05

**Authors:** Ramesh Omranipour, Sadaf Alipour, Maryam Hadji, Forouzandeh Fereidooni, Issa Jahanzad, Khojasteh Bagheri

**Affiliations:** 1 Cancer Research Center, Cancer Institute, Tehran University of Medical Sciences, Tehran, IR Iran; 2 Department of Surgery, Cancer Institute, Tehran University of Medical Sciences, Tehran, IR Iran; 3 Department of Surgery, Arash Women’s Hospital, Tehran University of Medical Sciences, Tehran, IR Iran; 4 Department of Pathology, Cancer Institute, Tehran University of Medical Sciences, Tehran IR Iran; 5 Department of Immunohistochemistry, Cancer Institute, Tehran University of Medical Sciences, Tehran, IR Iran; 6 Breast Clinic Cancer Institute, Tehran University of Medical Sciences, Tehran, IR Iran

**Keywords:** Breast, Neoplasm, Carcinoma, Estrogen, Progesterone

## Abstract

**Background:**

Diagnosis of breast cancer is completed through core needle biopsy (CNB) of the tumors but there is controversy on the accuracy of hormone receptor results on CNB specimens.

**Objectives:**

We undertook this study to compare the results of hormone receptor assessment in CNB and surgical samples on our patients.

**Patients and Methods:**

Hormone receptor status was determined in CNB and surgical samples in breast cancer patients whose CNB and operation had been performed in this institute from 2009 to 2011 and had not undergone neoadjuvant chemotherapy.

**Results:**

About 350 patients, 60 cases met all the criteria for entering the study. The mean age was 49.8 years. Considering a confidence interval (CI) of 95%, the sensitivity of ER and PR assessment in CNB was 92.9% and 81%, respectively and the specificity of both was 100%. The Accuracy of CNB was 98% for ER and 93% for PR.

**Conclusions:**

Our results confirm the acceptable accuracy of ER assessment on CNB. The subject needs further investigation in developing countries where omission of the test in surgical samples can be cost and time-saving.

## 1. Background

In modern practice, the diagnosis of breast cancer is completed through the histological exam of core needle biopsy (CNB) samples(1). One of the important issues in treatment planning is the hormone receptor (HR) status of the tumor which can be assessed by immunohistochemitry (IHC) on the CNB specimens (CNBS) or over the subsequent surgical sample (SS).When neoadjuvant therapies are necessary preoperatively, decision making would necessarily be based on the evaluation of the needle biopsy samples (2).

## 2. Objectives

The concordance of estrogen receptors (ER) and progesterone receptors (PR) on CNBS and SS is not fully documented. We undertook this study to compare the results of ER and PR in CNBS with those of SS in our patients.

## 3. Patients and Methods

Breast cancer patients attending the breast clinic of the Cancer Institute of Tehran University of Medical Sciences, Tehran, Iran, from October 2009 to October 2011 were considered for the cross sectional study. Those whose tissue diagnosis by CNB and operation had been performed in this institute were entered in the study. Patients who had received any form of neoadjuvant therapy were excluded to avoid probable effects of the treatment on the receptor status. Discordant histologies in CNBS and SS and cases with more than one tumor in one breast were excluded but bilateral cases whose HR status had been determined separately were included and regarded as 2 cases. Demographic features of the patients and their menstrual histories as well as the tumor histology, stage and grade of cancers, number of biopsies and volume of CNBSs were extracted from the records of the breast clinic. Those with missing data were excluded. Core needle biopsies were done by surgeons on palpable masses and by radiologists as ultrasound-guided or stereotactic biopsy in non-palpable cases. The surgeries included mastectomy or breast conserving surgery with or without oncoplasty; the axilla was managed by sentinel lymph node biopsy or axillary dissection based on lymph node status.

HR status had been determined in all cases on either the CNB or SS and the patients had been treated according to these. All the tests had been done by one experienced board-certified pathologist. In order to conduct our study, the sample which had not been investigated for receptor status was determined and the HR status was investigated by the same pathologist. All the samples had been fixed in 10% neutral buffered formalin, the CNBS for 6 hours or more and the SS for 24 hours. The pathologist reviewed the hematoxylin-eosin slides and selected paraffin blocks containing well preserved, non-necrotic tumors for IHC. IHC was performed using Clone 1D5 (Dako, Code N1575) anti-estrogen antibody for ER detection and clone PgR 636 (Dako, Code N1630) anti-progesterone antibody for PR and DAKO envision detection kit. Immunoreactivity of more than 1% of the tumoral cells was interpreted as a positive result. We assumed the IHC of the SS as the gold standard and CNB as the test; sensitivity, specificity, negative and positive predictive values of ER and PR of CNBS were then calculated considering a confidence interval (CI) of 95% for all values. STATA statistical software version 11.2 was used for statistical analyses. The Regional Ethics Committee of Tehran University of Medical Sciences approved this study on September 2009.

## 4. Results

About 350 cases of breast cancer attending the breast clinic in the period of the study, 60 cases met all the criteria for entering the study and included enough tumoral tissue to undergo IHC. Out Of these, 43 had their HR status determined in the CNBS and 17 in their SS. We thus performed the test on 43 CNBS and17 SS. The mean age of the patients was 49.8 ± 12.8 (Mean ± SD) years. The age and menopausal status of the patients, the tumor histology, stage and grade as well as the number of HR positive and negatives in each category in addition to the number of cores taken for one CNB and total volume of CNBSs are demonstrated in Table 1.

**Table 1.** Patients and Tumors Characteristics

	Number	Percent (%)
**Age**		
≤ 35	9	15
35-50	19	31.6
≥ 50	32	53.3
**Menopause Status**		
premenopausal	28	46.6
Menopause	32	53.3
**Tumor Histology**		
IDC^a^	56	93.3
ILC^a^	1	1.6
DCIS^a^	3	5
**Disease Stage^b^**		
0	2	3.3
I	5	8.3
II	47	78.3
III	6	10
IV	0	0
**Histologic Grade II^c^**		
1	7	11.6
2	35	58.3
3	15	25
IS	3	5
**Number of Specimens in CNB**		
1-3	1	1.6
4-6	34	56.6
> 6	25	41.6
**Overall Volume of CNBS (cm3)**		
< 0.02	4	6.6
0.02-0.04	7	11.6
≥ 0.04	49	81.6

^a^IDC, invasive ductal carcinoma; ILC, invasive lobular carcinoma; DCIS, ductal carcinoma in situ; IS, in situ cases

^b^Disease Stage, based on American Joint Committee on Cancer (AJCC) TNM system

^c^Histologic Grade

The ER status in SS was positive in 46 (76.6%) and negative in 14 (23.3%). The PR status in these samples was positive in 39(65%) and negative in 21 (35%). The ER status of the CNBS was positive in 47 (71.7%) and negative in 17 (28.3%) while the PR was positive in 43 (71.7%) and negative in 17(28.3%). In CNBS, there were 2 ER positive, PR negative tumors (3.33%); these were 6 in SS results (10%). There was no PR positive, ER negative tumor in either. Considering a confidence interval of 95%, the sensitivity of ER and PR assessment in CNB was 92.9% (66.1-99.8%) and 81 %( 58.1-94.6%) respectively. The specificity of both was 100% (92.3-100% for ER and 91-100% for PR, CI = 95%). The accuracy of CNB was 98% for ER and 93% for PR.

The positive and negative predictive value of ER and PR in CNBS (CI = 95%) was equal to 100% (75.3-100%), 97.9% (87.1-99.9%), 100% (80.5-100%) and 90.7% (77.9-97.4%), respectively. Overall, there were 4 discordant cases for PR, all but one was in stage II of the disease and the other had in situ carcinoma. Two of the patients were in our higher age group (> 50 years) and the other two in the second range (35-50 years). Discordance for ER was seen in a 44 years old lady with a stage II, grade 2 invasive ductal carcinoma. Two of the PR discordant cases had 4-5 cores taken for the biopsy, the 3 other discordants had at least 6 cores.

## 5. Discussion

Tissue diagnosis of breast cancer by CNB before proceeding to any kind of treatment is the gold standard of modern medical practice. The accuracy of histologic identification is superior to the cytologic diagnosis by fine needle aspiration and its costs are extremely lower than the old practice of excisional biopsy. Accordingly, physicians can discuss the treatment plan with the patient preoperatively and give her the chance of one-step operation. As well, neoadjuvant treatment, when indicated, can be based on the grade assessment and HR status of the CNBS. In cases of complete pathologic response, this will be the only source for evaluation of HR (1, 2). It can also determine the HR status more accurately than the IHC exam of an excised tumor with partial response to neoadjuvant chemotherapy.

More than two thirds of breast cancers are HR positive worldwide. False negative HR result leads to inaccurate management and erroneous omission of hormone therapy (3). Determination of HR as measured in CNBS is therefore very important and literature discusses the subject accordingly. In 1997, Zidan et al. (4) assessed the HR in 26 clinically palpable breast cancers including CNBS and excisional biopsy specimens. They interpreted their results as weakly positive, moderately positive or strongly positive. They then calculated the absolute agreement between scoring categories and the agreement after adding all categories together in CNBS and SS. The absolute and added agreement for ER and PR were respectively demonstrated as 73%, 93%, 42% and 69%. There was no case of ER negative CNB with ER positive excision in their study, which they attributed to more rapid and better fixation of the CNBS because of smaller volume. On the other hand, the less concordant PR results (compared to ER) were explained by a probable greater heterogeneity of these receptors in the cancerous tissue. Ten years later, a similar study found concordance rates of 95% and 89% for ER and PR respectively between 87 CNBS and excisional biopsy samples (5). From 2005 to 2007, Arnedos et al (2) retrospectively investigated the HR data of early breast cancers attending The Royal Marsden Hospital who had their tumors biopsied by core needles and then excised surgically; they found out that ER was concordant in 98.2% and PR in 85% in 336 pairs of samples.

Nonetheless, some studies have shown high levels of accuracy for PR estimation in CNBS; Park et al. (6) detected 99% and 97.1% concordance rates for ER and PR in CNBS and SS of104 invasive ductal cancers and Richter-Ehrenstein et al. (7) likewise detected a high rate of agreement for both ER and PR assays in 567 pairs of samples. Sutela et al. (8) reported higher concordance in PR results than ER between CNBS and SS in their 41 cases (83% for ER and 88% for PR) while discordance was mostly detected as positive HR in CNB with negativity in SS. The discordance of PR results in CNBS compared with SS was attributed to scarcity of biopsy numbers in CNB in the work of Tamaki et al. (9) (overall concordance rate of 92.9% for ER and 89.3% for PR in 353 cases) who showed a high agreement in PR of the two specimens when more than 3 cores were taken. However, Ough et al. (1) did not approve the case; concordance rate between CNB and SS for PR was only 78% (and 88% for ER) among their 209 breast cancers whereas 4-6 cores were taken for each CNB. They concluded that ER distribution is probably more homogenous in breast cancer, and claimed that treatment planning based on CNB HR results could be unsafe. Lorgis et al. (10) attempted to provide further precision by performing each test twice in CNBS and excised tumors in their 175 early breast cancers but revealed figures of 84% and 78.3% for concordance of ER and PR, respectively. They concluded that HR results of CNB should be used with caution. It is worth mentioning that Uy et al. (3) recommend using CNBS for assessment of HR status because, like Zidan et al. (4), they believe in a more appropriate fixation of the sample and less tumor ischemia due to smaller volumes of the specimens. HR positivity was more frequent in their CNB than SS (modified radical mastectomy in all of their cases), and they recommend to assess the SS only in case of doubtful processing of the CNBS, especially in HR negative results. There are two recently published meta-analysis on the subject. The pooled sensitivity calculated for ER and PR in the study of Chen et al. (11) was respectively 97% and 91%, and 97.3 % and 92.3% in the study of Li et al. (12) when comparing CNBS and SS. The authors of both papers conclude that the accuracy of the test in CNB is high but recommend checking the receptor status in both samples particularly in HR negative cases.

In our study, all of the discordant invasive cases were in stage II of breast cancer but this could not be evidence for any relation between these factors because of the misleading stage distribution in our study population due to exclusion of metastatic (stage IV) cases and those in need of neoadjuvant therapy (most of stage III patients). Our results confirm the acceptable accuracy of ER in CNB. On the other hand, ER results have a higher impact on treatment decisions and the results seem good enough to allow single checking of HR on CNBS. Our suggestion is to confirm the negative results in CNBs on the surgical specimen. We believe that this subject needs yet further investigation, particularly in developing countries where the cost of double checking is high, and omission of the test in SS ( when it has been checked on CNBS) is cost-saving. Then again, it is in these areas that the completion and interpretation of specialized laboratory tests such as IHC assays may take long enough to threaten the golden time for best treatment. Time could definitely be saved if the postoperative adjuvant therapies could be based on CNBS HR results.
